# Clinical challenges in the management of cardiac amyloidosis complicating aortic stenosis and coronary artery disease

**DOI:** 10.3389/fcvm.2022.1061717

**Published:** 2022-12-12

**Authors:** Maharshi Raval, Sajid Siddiq

**Affiliations:** ^1^Department of Internal Medicine, New York Medical College, Valhalla, NY, United States; ^2^Department of Internal Medicine, Landmark Medical Center, Woonsocket, RI, United States; ^3^Department of Cardiology, New York Medical College, Valhalla, NY, United States; ^4^Department of Cardiology, Landmark Medical Center, Woonsocket, RI, United States

**Keywords:** cardiac amyloidosis, coronary artery disease, aortic stenosis, TAVR, PCI, PYP scan

## Background

In 2017, Castano et al. studied 151 patients undergoing transcatheter aortic valve replacement (TAVR) for severe symptomatic aortic stenosis (AS) to determine the prevalence of cardiac amyloidosis (CA) and found 16% of patients to have concomitant ATTR-CA and AS. This opened the question about the role of screening for ATTR-CA with the help of a quick and easy yet confirmatory 99mTc-PYP scan as a part of the pre-TAVR evaluation. Five years later, the cardiology community still has no specific guidelines in the area (1).

Another question that comes with such findings is the role of TAVR in managing such patients with aortic stenosis. The question of SAVR vs. TAVR in these cases is now redundant, with the benefit of TAVR being established in such high-risk cases. Considering the risk of surgical intervention, it was postulated that risk might also be high in a patient undergoing TAVR, and an argument for medical therapy over intervention was made.

Nitsche et al. studied 407 with concomitant CA and AS and found no worsening of outcomes with TAVR compared to patients with lone AS without CA. Results from the study of Rosenblum et al. were similar in terms of mortality, however, did note increased heart failure hospitalization after TAVR in patients who had ATTR-CA and AS (2, 3). There are limitations to these studies regarding patient selection. Patients were screened based on clinical scores, and a high-risk group of paradoxical low-flow, low-gradient AS was not considered. Data regarding the optimum timing of screening and management of CA in patients being evaluated for TAVR are lacking.

Coronary artery disease (CAD) management in patients with cardiac amyloidosis is another unexplored subject. There have been no large-scale studies to look for peri-procedural complications of percutaneous coronary intervention (PCI) in patients with CA. The question in this study is not about patients presenting with acute coronary syndrome (ACS) but rather about patients in the community with angina and equivocal stress test results. No studies adequate to help create guidelines for severity-based management are currently available.

## Discussion

Major studies focusing on patients with CA-AS compared to lone AS undergoing TAVR have shown a significant prevalence of CA in these patients. This ranges from 8.4 to 16% in various studies (1–4). However, it is estimated that this number may be as high as 30% in patients with paradoxical low-flow, low-gradient AS (5). Given such a high prevalence to be noted in studies and estimated by experts, the question is whether patients with severe AS being referred for TAVR benefit from routine screening for ATTR CA.

There have been significant advances in therapeutic strategies for ATTR CA. TTR stabilization is perceived to be a significant player in this territory. Results from clinical trials related to tafamidis have shown considerable mortality and morbidity benefit. Thus, it is recommended that therapy should be instituted as soon as the diagnosis of ATTR-CA is established (5). With data available regarding the high prevalence of ATTR-CA in patients with AS and the demonstration of effective treatment strategies, we opine that all patients undergoing TAVR should be routinely evaluated for ATTR CA with the help of a 99mTc-PYP scan after ruling out AL amyloidosis.

TTR gene silencer therapies have shown promising results for the management of amyloid neuropathies, however, have not been studied for the management of cardiac amyloidosis. It is only a matter of time before medications such as patisiran, inotersen, and vutrisiran are studied, and with their mechanism of action, they will likely show further benefit. If appropriate screening guidelines are established at this point, the medical community and the patients will only reap the benefits later when therapies become available and are more cost-effective.

Even if screening guidelines are framed and applied, the dilemma does not end in this study. A question that remains is how we manage such patients found through screening. As already discussed, based on current evidence, they will need to be started on TTR stabilizer therapy. But when should these patients undergo valve intervention? Guidelines based on a severity-based approach are lacking in this area as well. If a patient presents with decompensated AS, who is symptomatic, and hospitalized due to heart failure deemed secondary to AS, it is understandable for them to undergo TAVR earlier than later, likely while inpatient. Data from studies also back this by showing that TAVR is not associated with increased mortality in patients with CA-AS compared to lone AS.

The dilemma is most significant for the patients in the outpatient community with severe AS, although relatively stable. The currently available studies have enrolled patients referred for TAVR but randomized before heart-team evaluation. Although it appears to show the safety of TAVR, significant and convincing data are only available in terms of mortality. Morbidity data are scarce but do show an increased risk of complete heart block requiring permanent pacemaker implantation, increased heart failure symptoms, and hospitalizations (3, 6). In patients with non-critical AS with ATTR CA, a trial of TTR stabilizer or TTR silencer therapy could be considered before outpatient TAVR. The rationale is to medically optimize the patient before intervention and decrease morbidity associated with the procedure. Future randomized controlled trials are indicated with the treatment arm comprising patients receiving TAVR; however, after a trial of disease-modifying therapy, and the control arm comprising patients without any pre-treatment. If such a study shows improved morbidity, subsequent studies should aim at finding the optimum duration of such a trial therapy.

The era of emerging therapies for ATTR CA and concomitant relatively stable subacute and stable CAD is an area that is devoid of any significant research. Relatively stable CAD, diagnosed on functional perfusion study or coronary computed tomography angiogram (CTA), may be felt to be causing diastolic congestive heart failure (CHF) or mild ischemic cardiomyopathy (CMP). Clinicians in this setting would frequently resort to PCI and sometimes surgical revascularization to alleviate the perceived symptoms, only to be surprised post revascularization when there is no improvement. Sometimes, patients may actually deteriorate. The underlying problem may be ATTR CA, and CAD may be an innocent bystander. Case reports have shown that cardiac amyloidosis may present with recurrent angina and give a clinical appearance similar to CAD (7, 8). However, studies determining the prevalence of CA in patients with CAD are not performed yet. Amyloidosis has been postulated to result in CAD by depositing amyloid fibrils contributing to atherosclerosis (9). Despite this, as no data are available on this matter, we do not believe routine screening for ATTR CA in all patients with CAD may be necessary. However, in the presence of any of the red flag signs suggestive of CA, a 99mTc-PYP scan should be pursued.

Treatment of concomitant CAD and CA is not straightforward either. No studies have been explicitly performed looking at immediate complications of PCI, such as coronary artery perforation, coronary artery dissection, coronary artery aneurysm, failure of stent deployment, and patient-stent mismatch in patients with CA when compared to patients without CA. Immediate attention may be needed in this matter, and studies need to be performed looking at this research question as if it shows any role of CA in PCI complications; an argument can then be made to treat and stabilize the patients with disease-modifying therapy before PCI for stable angina. It may even be possible that with treatment, we may see significant improvement to the extent that patients may not even need intervention as symptoms may be primarily secondary to CA in such patients rather than CAD.

## Conclusion

The presence of underlying ATTR CA in patients with severe AS results in a tenuous clinical state associated with low stroke volume and elevated filling pressures. Heart failure and resultant morbidity, pre and post-intervention, remains. The jury is out on mortality. Whether disease-modifying therapy for ATTR CA could help alleviate AS-related morbidity remains to be seen. Similarly, these therapies may have a role before revascularization as well. The answer to many of these questions will come from adequately powered randomized studies.

## Author contributions

MR drafted the manuscript under the guidance of SS, who also reviewed the article for intellectual content. All authors contributed to the article and approved the submitted version.
